# Polarization-Independent Metasurface Color Filter with Side-Peak Suppression in Metallic Nanohole Array

**DOI:** 10.3390/s26082339

**Published:** 2026-04-10

**Authors:** Hui-Jin Yun, Seung-Yeol Lee

**Affiliations:** School of Electronic and Electrical Engineering, College of IT Engineering, Kyungpook National University, Daegu 41566, Republic of Korea; hj.sonar18@knu.ac.kr

**Keywords:** plasmonic color filter, extraordinary optical transmission, nanoaperture array

## Abstract

Recent advances in metasurface-based research have enabled significant reductions in the size and weight of optical devices. By employing metallic nanostructures with subwavelength dimensions, color filtering can be achieved through phenomena such as extraordinary optical transmission (EOT), which allows specific bands of visible light to pass through. However, conventional EOT-based color filters often suffer from strong side peaks outside the desired transmission band, degrading color purity and hindering accurate color reproduction. In this study, we propose an ultrathin, polarization-independent color filter based on a nanohole array that utilizes the EOT effect while effectively suppressing unwanted side peaks. To achieve this, we introduce a modified design in which additional metallic triangular edges are placed around a hole in a conventional hole array. This configuration suppresses higher-order diffraction modes and enables selective transmission at RGB wavelengths, thereby improving spectral selectivity and overall color performance.

## 1. Introduction

Color filters are optical components that selectively transmit or reflect specific wavelengths of light to produce desired colors. They are widely utilized in imaging sensors, organic light-emitting diode displays (OLEDs), and liquid crystal displays (LCDs). Conventional color filters are typically fabricated using pigments or synthetic dyes, which operate by absorbing certain wavelengths while transmitting others [1]. However, these dye-based organic filters suffer from several drawbacks: they are sensitive to heat and ultraviolet radiation, raise environmental concerns, and are difficult to scale to ultra-high resolution displays due to their relatively large fabrication feature sizes, which hinder their integration into compact optical systems. To address these limitations, structural color filters have emerged as a promising alternative. These filters utilize nanostructures that interact with light through optical phenomena such as diffraction, interference, and scattering, thereby producing tunable colors [2,3,4]. Structural color filters can be engineered using thin films [5], metamaterials [6,7], and quantum dots [8], offering enhanced stability and resistance to fading owing to their structure-dependent coloration mechanisms.

Among various structural color filter technologies, metasurface-based color filters have attracted particular attention [6,9]. A metasurface is an ultrathin optical structure composed of nanoscale unit cells whose dimensions are comparable to or smaller than the wavelength of incident light [10]. Despite their subwavelength thickness—typically on the order of tens to hundreds of nanometers—metasurfaces can replicate and even surpass the optical functionalities of conventional bulk optical elements. The high design flexibility afforded by geometry and arrangement of meta-atoms enables the precise manipulation of light properties such as reflection, refraction, and diffraction. Consequently, metasurface color filters offer highly tunable spectral responses, ultrathin form factors, and submicron pixel sizes, making them well-suited for next-generation photonic devices, including ultra-high resolution displays, holographic imaging, and augmented reality (AR) systems.

In particular, surface plasmon-based (SP-based) color filters have emerged as a promising approach, as they can manipulate light at subwavelength scales through the excitation of surface plasmon resonances [11]. However, most existing structural color filters relying on surface plasmonic resonances are inherently reflective [12,13,14], which greatly limits their applicability in transmissive optical systems. This limitation primarily arises from the intrinsic ohmic losses of metals, which severely reduce transmission efficiency and degrade color purity. Only a few transmissive SP-based color filters have been reported, and these are typically subtractive color filters (cyan, magenta, and yellow) [15,16] or suffer from low color purity and chromatic performance [17,18,19,20]. Consequently, the development of transmissive additive color filters (red, green, and blue) with high saturation and wide color gamut is, therefore, required for next-generation imaging sensors and display technologies.

By employing metallic nanostructures with subwavelength dimensions, color filtering can be achieved through phenomena such as extraordinary optical transmission (EOT), which selectively transmits specific bands of visible light [21,22]. Because the transmission characteristics can be readily tuned by adjusting the size and periodicity of the nanoholes, EOT-based color filters have been widely investigated and applied [23,24,25]. However, conventional EOT-based color filters often suffer from undesirable side peaks outside the primary transmission band. Due to the finite size of the nanostructures, light scattering from the nanohole arrays cannot fully suppress higher-order diffraction modes, resulting in significant color crosstalk. Such spectral interference degrades color saturation and reduces the achievable color gamut, thereby limiting practical applicability. Therefore, to satisfy the requirements for high-quality color rendering, it is highly desirable to design EOT-based color filters with enhanced monochromaticity.

In this study, we propose an ultrathin, polarization-independent color filter based on a metallic nanohole array that exploits the EOT effect while effectively suppressing undesired side peaks. To accomplish this, we introduce a modified structural design in which additional metallic triangular edges are incorporated around a nanohole array. This configuration efficiently suppresses higher-order diffraction modes and enables selective transmission at red, green, and blue (RGB) wavelengths, thereby improving spectral selectivity and enhancing its overall color performance. Owing to its nanostructured architecture, the proposed filter is significantly thinner than conventional organic color filters, providing advantages in integration and miniaturization. Furthermore, the design exhibits polarization insensitivity, making it well-suited for integrated photonic devices and next-generation display technologies, such as white organic light-emitting diode on silicon (WOLEDoS) systems. In addition, because the proposed color filter consists of a single metallic layer, it can be readily incorporated into cavity-based configurations, providing design flexibility for a wide range of optical and optoelectronic applications.

## 2. Design of the Color Filtering Metasurface

**Figure 1:**
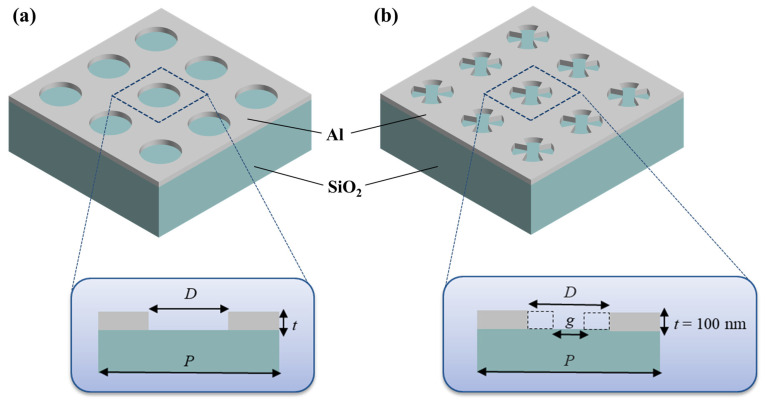
Schematics of (**a**) a conventional nanohole array and (**b**) proposed color filtering metasurface.

Figure 1 illustrates the schematics of a conventional nanohole array (Figure 1a) and the proposed side-peak suppressed color-filtering metasurface (Figure 1b). In both designs, the apertures are patterned in a 100 nm-thick (*t*) aluminum (Al) layer deposited on a SiO_2_ substrate. The proposed color filter incorporates symmetrically arranged metallic triangular edges embedded within each nanohole. The geometrical parameters are defined as follows. The conventional nanohole array is fully characterized by its period (*P*) and hole diameter (*D*). In contrast, the proposed structure introduces additional design parameters, including the inner gap between two opposing nanoedges (*g*) and apex angle of triangular edge (θ), which must be considered to accurately describe the modified geometry.

Throughout the manuscript, three-dimensional (3D) finite-difference time-domain (FDTD) simulations were performed to investigate the optical properties of the proposed structure. In the simulations, the light source was modeled as a normally incident plane wave with polarization oriented along the x-axis. For the metasurface geometry, periodic boundary conditions were applied along the x- and y-directions, while perfectly matched layer (PML) boundary conditions were employed along the z-axis to eliminate unwanted boundary reflections. The electric permittivity of aluminum was taken from the experimental data reported by Palik [26]. The medium above the Al layer was assumed to be air. After that, the electric field distributions and transmission spectra were analyze.

The primary mechanism underlying extraordinary optical transmission (EOT) is the interaction of surface plasmon polaritons (SPPs) supported by nanohole arrays [27]. SPPs are excited when incident light couples to the collective oscillations of free electrons at the metal–dielectric interface, providing additional momentum that enables the conversion of the incident electromagnetic wave into a surface plasmon on a metallic film perforated with subwavelength holes. The dispersion relation of the SPPs can be derived from Maxwell’s equations under appropriate boundary conditions [28,29]. The wavelength-dependent SPP wave vector *k* can be expressed as follows,

(1) $$\begin{array}{c} \begin{array}{c} k=k_{0}\sqrt{\frac{\varepsilon_{m}\varepsilon_{d}}{\varepsilon_{m}+\varepsilon_{d}}} \end{array} \end{array}$$

where $\varepsilon_{m}$ and $\varepsilon_{d}$ are the permittivity of the metal and the dielectric medium, respectively. *k*_0_ represents the wave vector in free space. Owing to the excitation of SPPs, optical transmission through a metallic nanohole array becomes significantly higher than that predicted by conventional diffraction theory, such as Bethe’s theory [30,31]. The SPPs interact with evanescent waves scattered at the metal–air interface after passing through the nanoholes, thereby enhancing the portion of light that propagates into the far field and resulting in extraordinary transmission. Accordingly, under normal incidence, the transmission peaks originate from SPP modes supported by a two-dimensional lattice, which can be described by the following dispersion relation,

(2) $$\begin{array}{c} \begin{array}{c} \lambda_{res}\left(i,j\right)=\frac{P}{\sqrt{i^{2}+j^{2}}}\sqrt{\frac{\varepsilon_{m}\varepsilon_{d}}{\varepsilon_{m}+\varepsilon_{d}}} \end{array} \end{array}$$

Here, *i* and *j* represent the diffraction orders of the array, which determine the propagation direction of the SPPs. As indicated by Equation (2), the resonance wavelength is governed by the structural parameters of the nanostructure, including the periodicity of the hole array, the optical properties of the metal film, and the surrounding dielectric medium. In addition to propagating SPP modes, surface plasmons can also exist in the form of localized surface plasmons (LSPs), which enhance the local electric field at the edges of the subwavelength holes. These LSPs couple with the SPPs, further contributing to the enhancement of optical transmission. Together, these plasmonic modes—both SPPs and LSPs—constitute the fundamental mechanism underlying EOT, and by carefully engineering and controlling these modes, an ultrathin color filter based on metallic nanohole arrays was proposed. In the proposed work, the addition of triangular edges can enhance the LSPs under specific wavelength conditions, which can be exploited to effectively suppress the side-peaks observed in conventional nanohole arrays.

## 3. Results and Discussion

**Figure 2:**
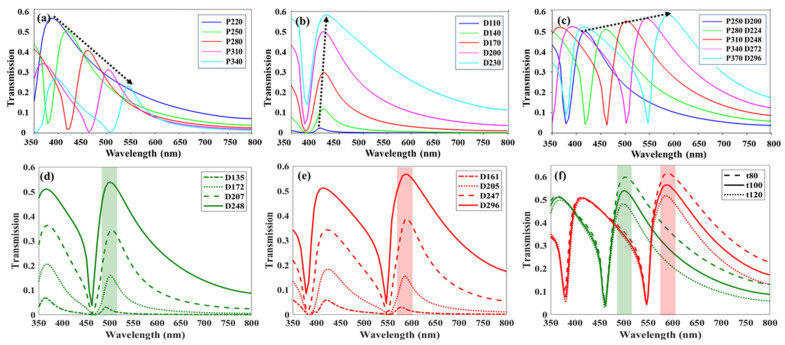
Structural parameter optimization of the nanohole array. (**a**) Redshift characteristics of transmission resonance as the *P* varies with a fixed condition of *D* = 200 nm. (**b**) Variation in transmission spectra with changing *D* under a fixed *P* = 250 nm. (**c**) Trend of redshift when the diameter-to-period ratio is fixed at 1:1.25 and the overall structural dimensions are scaled. (**d**) Green (*P* = 310 nm) and (**e**) red (*P* = 370 nm) bands. The diameter sweeps at fixed periods, which illustrate the optical responses as the diameter-to-period ratio changes in the green and red bands, respectively. (**f**) Optimization of the Al film thickness (*t*) for the selected green (*P* = 310 nm, *D* = 248 nm) and red (*P* = 370 nm, *D* = 296 nm) configurations.

First, we investigated the influence of the key structural parameters—the *P* and *D*—on the EOT characteristics of a simple metallic nanohole array. The dashed arrows in Figure 2 indicate the shifts of the transmission resonance peaks as the structural parameters are varied. As shown in Figure 2a, when the hole diameter *D* is fixed at 200 nm, and the period *P* is increased from 220 nm to 340 nm in increments of 30 nm, the dominant transmission peak wavelength (*λ*_res_) exhibits a redshift from 430 nm to 560 nm. This redshift is accompanied by a reduction in overall transmittance and a narrowing of the full width at half maximum (FWHM). These trends can be explained by the dispersion relation of surface plasmons in the two-dimensional (2D) lattice, as described in Equation (2). In Figure 2b, the period *P* is fixed at 250 nm, while the hole diameter *D* is varied from 110 nm to 230 nm in steps of 30 nm. In this case, the resonance wavelength remains nearly unchanged, whereas the overall transmission intensity increases with larger hole diameters. This behavior is attributed to the increased aperture area ratio of the nanohole array.

Based on these observations, we established a design strategy to precisely position the resonance peaks at RGB wavelengths. Increasing the aperture-to-period ratio was found to enhance transmittance but simultaneously broaden the FWHM, exhibiting a trade-off between transmission efficiency and spectral selectivity. To balance these competing factors, the diameter-to-period ratio was fixed at 1:1.25, and the overall structure sizes were scaled to tune the resonance positions to the desired RGB wavelength bands, as illustrated in Figure 2c. Specifically, the red filter was designed with *P* = 370 nm and *D* = 296 nm, while the green filter employed at *P* = 310 nm and *D* = 248 nm, providing an optimal balance between transmission strength and bandwidth. Accordingly, these optimized configurations are selected for the following investigations, such as the addition of triangular edges.

The rationale behind selecting these specific parameters is supported by the detailed diameter sweeps shown in Figure 2d,e for the target green (*P* = 310 nm) and red (*P* = 370 nm) bands, respectively. The selected diameters (indicated by solid lines) provide the optimal balance between transmission strength and bandwidth. Furthermore, the optimization of the Al film thickness (*t*) was investigated, as presented in Figure 2f. We examined the transmission spectra at thicknesses of *t* = 80, 100, and 120 nm. As anticipated, a thinner film of 80 nm enhances the EOT peak but leads to a broadened FWHM, thereby degrading color purity. Conversely, at *t* = 120 nm, the overall transmittance decreases due to the increased attenuation in the thicker metallic layer. Consequently, *t* = 100 nm (solid lines) was determined to provide the most optimal balance, and was, thus, maintained for the designs. Ultimately, the design of the proposed structure relies on strategically managing the trade-off between the FWHM and transmittance. The specific prioritization of these two competing factors can be flexibly adjusted depending on the specific brightness or color fidelity requirements of target practical applications.

**Figure 3:**
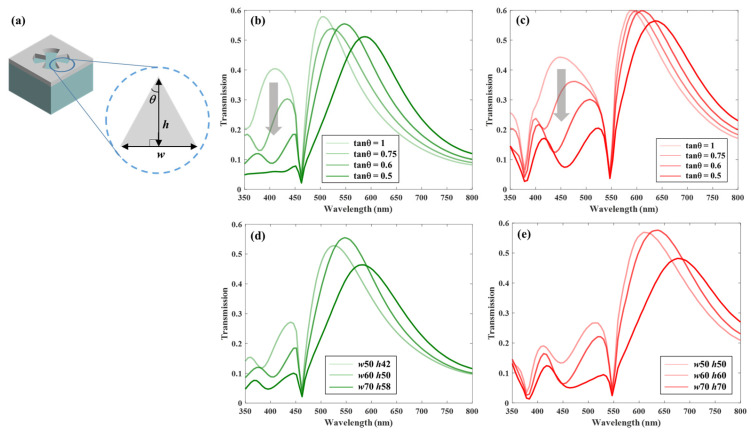
Transmission characteristics and geometric optimization of the edge-modified nanohole arrays for the green and red color filters. (**a**) Definition of the structural parameters of the triangular edge. Transmission spectra of (**b**) the green color filter and (**c**) the red color filter as a function of tanθ, with the fixed width at *w* = 60 nm. As the apex angle decreases, stronger suppression of the side peaks is observed, as indicated by the thick gray arrows. Transmission spectra of (**d**) the green color filter and (**e**) the red color filter as a function of the overall size (*w* and *h*) of the triangular edges, with the fixed tanθ at 0.6 and 0.5, respectively.

Figure 3 demonstrates the suppression of side peaks achieved by incorporating metallic triangular edges around a hole in a simple hole array, which is designed with appropriate *D* and *P* conditions based on Figure 2. The degree of side-peak suppression depends strongly on the size and geometry of the triangular edges. Figure 3a presents a schematic illustrating the width (*w*), height (*h*), and angle (*θ*) of the triangular edges, which are used to quantify edge sharpness. Here, the value of *w* is fixed at 60 nm to prevent excessive reduction of the aperture ratio as the edge size increases. The sharpness increases as the value of $\tan\theta =\frac{w}{2h}$ decreases.

Figure 3b,c presents the transmission spectra of the green and red color filters, respectively. As discussed previously, a simple nanohole array exhibits one dominant resonance peak accompanied by a secondary side peak. To suppress the side peaks, triangular edges are introduced to form narrow gaps within the nanohole array. This structural modification results in a single dominant transmission peak originating from resonant modes that are strongly confined within the gaps. It is observed that decreasing the value of tanθ leads to a more effective suppression of the side peaks. Furthermore, an additional optimization was performed in Figure 3d,e by varying the overall size of the triangular edges while maintaining the selected tanθ value (i.e., fixed sharpness), the green color filter with 0.6, and the red color filter with 0.5. The results indicate that larger triangular edges lead to a more pronounced suppression of the side peaks. However, a trade-off exists between side-peak suppression and the FWHM of the dominant resonance peak. In addition, the incorporation of the triangular edges induces a slight redshift in the resonance wavelength. The structural parameters were finely optimized using the parameter sweep in the Lumerical FDTD simulation.

Based on this optimization, the green color filter was designed with *w* = 60 nm and *h* = 50 nm (*g* = 148 nm), while the red color filter employed *w* = 60 nm and *h* = 60 nm (*g* = 176 nm), respectively. On the other hand, a simple nanohole geometry with *P* = 250 nm and *D* = 200 nm is employed for a blue color filter, as it does not exhibit side peaks within a visible wavelength, as shown by the blue curve in Figure 2c. The designed array is 4-fold symmetric, yielding identical optical characteristics for both *x*- and *y*-polarizations. Consequently, the proposed structure exhibits polarization-independent behavior, ensuring a consistent optical response for arbitrary polarization states.

**Figure 4:**
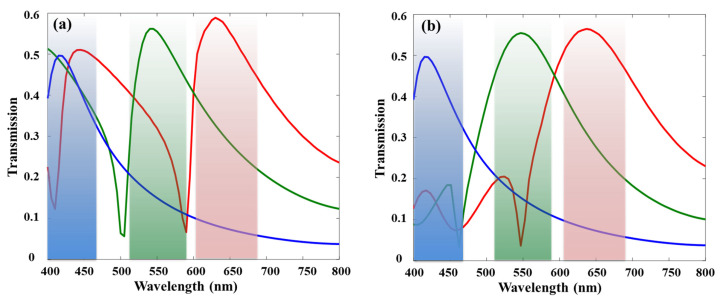
Transmission spectra of (**a**) the conventional nanohole array and (**b**) the proposed color filter incorporating triangular edges. The wavelength regions corresponding to the RGB colors are highlighted in their respective colors.

Figure 4 presents the transmission spectra of the blue, green, and red color filters under normal incidence for a conventional nanohole array (Figure 4a) and the proposed triangular-edge-added color filter (Figure 4b). As discussed previously, the conventional nanohole array with *P* = 250 nm and *D* = 200 nm was adopted for both cases of blue filter designs. In contrast, the green and red color filters exhibit a pronounced suppression of side peaks, particularly in the 400–450 nm range for green and the 400–550 nm range for red. This effective elimination of undesired resonance modes indicates that the proposed design provides improved monochromaticity and higher color saturation compared to the simple nanohole array, which typically supports multiple resonant modes. Moreover, the transmission efficiency at the resonance wavelengths exceeded 50% for all RGB color channels, demonstrating the sufficiently high transmittance achievable.

**Figure 5:**
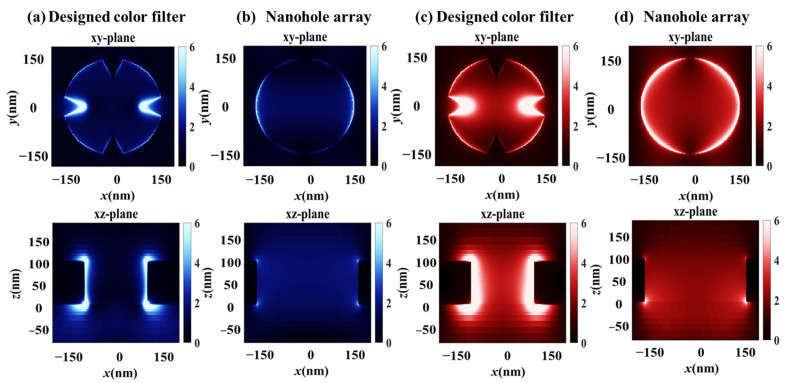
E-field distributions of the designed color filter and nanohole array along the *xy*-plane and *xz*-plane monitored at the wavelength of (**a**,**b**) 450 nm and (**c**,**d**) 650 nm.

To investigate the physical mechanism of the designed color filter, the electric field (E-field) distributions of a single unit cell were examined for the proposed red color filter and a simple nanohole array with *P* = 400 nm and *D* = 320 nm, using *xy*-plane and *xz*-plane monitors. The *xy*-plane monitor was positioned at the metal–dielectric interface. Figure 5a,b shows the E-field distributions at a wavelength of 450 nm, corresponding to the side-peak region. As shown in Figure 5a, for the proposed red color filter, strong localized surface plasmons (LSPs) are excited around the metallic triangular pillars, while surface plasmon coupling between adjacent apertures is significantly suppressed, leading to a reduced optical transmission. These LSPs are highly localized and do not effectively mediate inter-hole coupling; instead, they confine the electromagnetic energy locally and primarily contribute to absorption rather than transmission enhancement. Figure 5c,d presents the E-field distributions at the main resonance wavelength of 650 nm. In the *xz*-plane, a strong vertical cavity resonance is excited within the nanoholes, which dominates the transmission response despite the presence of strongly coupled LSPs. Consequently, both the proposed color filter and the conventional circular nanohole array exhibit high transmission at the dominant resonance wavelength, confirming that the primary transmission peak mainly originates from the vertical resonance within the apertures.

**Figure 6:**
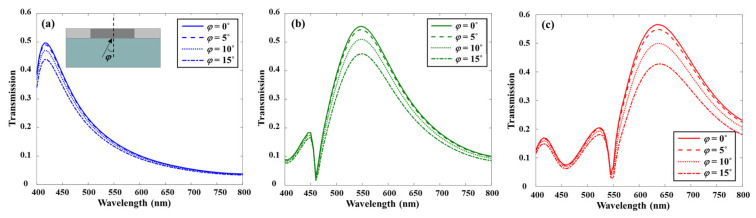
Transmission spectra of the proposed color filter designed for (**a**) blue, (**b**) green, and (**c**) red wavelength for different incident angles *φ*.

Figure 6 illustrates the relationship between the incident angle and the resonance wavelength for the blue, green, and red color filters. As the incident angle increased from 0° to 15°, only a slight reduction in transmission intensity was observed. Moreover, the resonance peaks for each color remain nearly unchanged, centered at approximately 450 nm, 550 nm, and 650 nm, respectively. The variation in the FWHM over this angular range is negligible, indicating minimal degradation in color purity. These results confirm that the proposed color filter exhibits high optical transmission characteristics and maintains stable spectral responses and consistent color performance under oblique light incidence.

**Figure 7:**
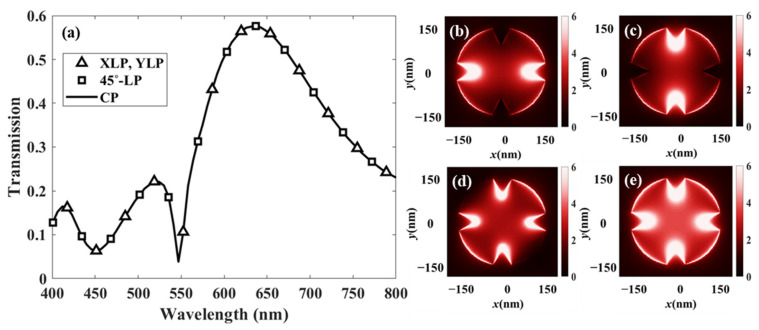
Polarization-independent transmission characteristics of the proposed red color filter at a wavelength of 630 nm under normal incidence. (**a**) Transmission spectra under linearly polarized light (x-, y-, and 45–polarizations) and circularly polarized light. E-field distributions under (**b**) x-linearly polarized light, (**c**) y-linearly polarized light, (**d**) 45–linearly polarized light, and (**e**) circularly polarized light.

To verify the polarization-independent operation of the proposed color filter, the transmittance of the red filter was characterized at a wavelength of 630 nm under normal incidence of plane waves with various polarization states, including Figure 7b–d linear polarization (x-, y-, 45–polarized each) and Figure 7e circular polarization. The structural symmetry of the unit cell was deliberately maintained to ensure polarization insensitivity, which is particularly advantageous for practical display and sensing applications.

As shown in the results, identical transmission spectra were obtained irrespective of the incident polarization state. The transmittance at 630 nm remained unchanged under tilted linear and circular polarizations, confirming the polarization-independent response of the proposed structure.

**Figure 8:**
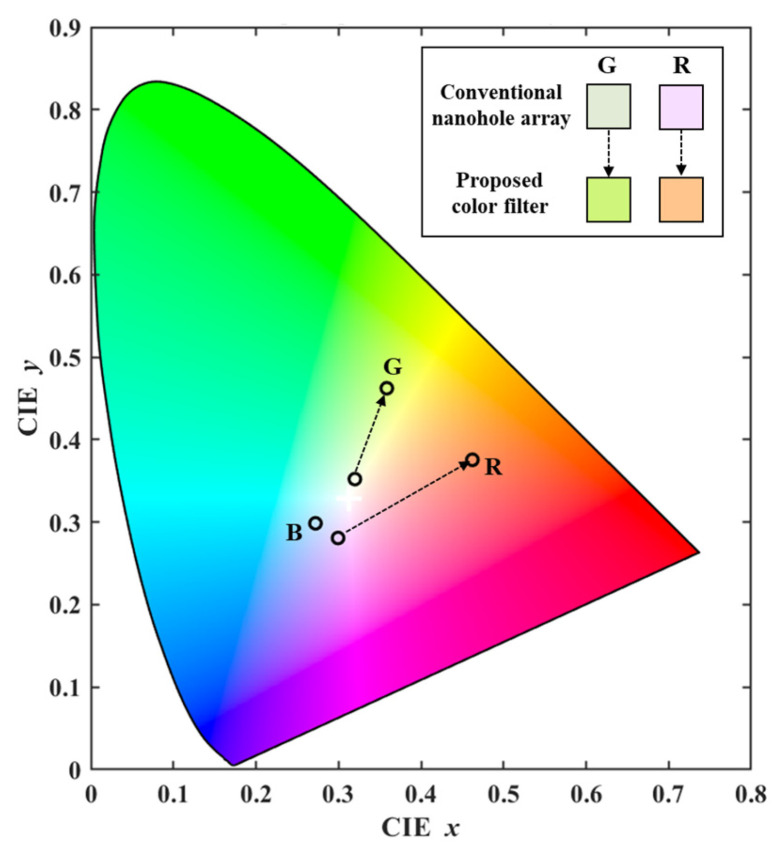
CIE 1931 chromaticity diagram the comparing conventional nanohole array and the proposed color filters.

To quantitatively evaluate the impact of side-peak suppression on the color filtering performance, the CIE 1931 chromaticity coordinates (*x*, *y*) were calculated from the simulated transmission spectra $T\left(\lambda \right)$. The CIE *XYZ* tristimulus values were obtained using the CIE 1931 2° standard observer color-matching functions $\bar{x}(\lambda )$, $\bar{y}\left(\lambda \right)$, and $\bar{z}\left(\lambda \right)$ over the visible wavelength range (400 nm to 800 nm) under the standard illuminant D65, expressed as [32,33]:

(3) $$\begin{array}{c} \left\{\begin{array}{c} X=k\int I\left(\lambda \right)T\left(\lambda \right)\bar{x}\left(\lambda \right)d\lambda \\ Y=k\int I\left(\lambda \right)T\left(\lambda \right)\bar{y}\left(\lambda \right)d\lambda \\ Z=k\int I\left(\lambda \right)T\left(\lambda \right)\bar{z}\left(\lambda \right)d\lambda \end{array}\right. \end{array}$$

where *I*(λ) denotes the spectral power distribution of the CIE standard illuminant D65, and the normalizing factor *k* is defined as 100/$\int I\left(\lambda \right)\bar{y}\left(\lambda \right)d\lambda$. Figure 8 illustrates the plotted positions of the conventional nanohole array and the proposed color filter on the CIE 1931 chromaticity diagram. The coordinates (*x*, *y*) were derived from the tristimulus values (*X*, *Y*, and *Z*), calculated using Equation (3), and normalized according to the following CIE 1931 standard [32,33]:

(4) $$\begin{array}{c} x\;=\;\frac{X}{X+Y+Z},\;\;y=\;\frac{Y}{X+Y+Z}\; \end{array}$$

The extracted CIE (*x*, *y*) coordinates for the proposed green and red filters are (0.359, 0.462) and (0.462, 0.375), respectively. In direct comparison with the conventional nanohole arrays, the proposed color filter exhibits a shift towards the spectral locus. This shift indicates that the strategic suppression of undesired sidebands yields an overall enhancement in color saturation and purity. Therefore, the geometric tuning of the nanoholes provides a viable approach to optimizing the color gamut for practical color-filtering applications.

Furthermore, it is worth noting that the underlying mechanism of suppressing side-peaks through geometric edge modification holds broader significance beyond color filtering applications. We anticipate that this strategic tuning of hole geometries can be adopted in various other hole-based nanostructures governed by the EOT phenomenon.

## 4. Conclusions

In this study, we designed and optimized an ultrathin metasurface color filter as a potential alternative to conventional organic-based color filters. The optimized structure demonstrates significantly improved color purity and transmittance compared to traditional nanohole-based designs, while showing an optical performance under incident polarization angle by maintaining a symmetrical structure along the *x* and *y* direction. Future work will focus on evaluating the scalability of the metasurface fabrication process for large-area applications, as well as conducting further optimization to achieve enhanced optical performance.

## Data Availability

The original contributions presented in this study are included in the article. Further inquiries can be directed to the corresponding author.
